# Declined miR‐181a‐5p expression is associated with impaired natural killer cell development and function with aging

**DOI:** 10.1111/acel.13353

**Published:** 2021-03-29

**Authors:** Jiao Lu, Shan Li, Xiaopeng Li, Wenming Zhao, Xuefeng Duan, Xiuling Gu, Jianqiao Xu, Bolan Yu, Luis J. Sigal, Zhongjun Dong, Lixin Xie, Min Fang

**Affiliations:** ^1^ CAS Key Laboratory of Pathogenic Microbiology and Immunology Institute of Microbiology Chinese Academy of Sciences Beijing China; ^2^ University of Chinese Academy of Sciences Beijing China; ^3^ Key Laboratory for Major Obstetric Diseases of Guangdong Province The Third Affiliated Hospital of Guangzhou Medical University Guangzhou China; ^4^ Department of Respiratory Medicine Chinese PLA General Hospital Beijing China; ^5^ Department of Microbiology and Immunology Thomas Jefferson University Philadelphia PA USA; ^6^ School of Medicine Tsinghua University Beijing China; ^7^ International College University of Chinese Academy of Sciences Beijing China

**Keywords:** aging, development, function, MiR‐181a‐5p, MiRNome, NK cells

## Abstract

MicroRNAs (miRNAs) regulate gene expression and thereby influence cell development and function. Numerous studies have shown the significant roles of miRNAs in regulating immune cells including natural killer (NK) cells. However, little is known about the role of miRNAs in NK cells with aging. We previously demonstrated that the aged C57BL/6 mice have significantly decreased proportion of mature (CD27^−^CD11b^+^) NK cells compared with young mice, indicating impaired maturation of NK cells with aging. Here, we performed deep sequencing of CD27^+^ NK cells from young and aged mice. Profiling of the miRNome (global miRNA expression levels) revealed that 49 miRNAs displayed a twofold or greater difference in expression between young and aged NK cells. Among these, 30 miRNAs were upregulated and 19 miRNAs were downregulated in the aged NK cells. We found that the expression level of miR‐l8la‐5p was increased with the maturation of NK cells, and significantly decreased in NK cells from the aged mice. Knockdown of miR‐181a‐5p inhibited NK cell development in vitro and in vivo. Furthermore, miR‐181a‐5p is highly conserved in mice and human. MiR‐181a‐5p promoted the production of IFN‐γ and cytotoxicity in stimulated NK cells from both mice and human. Importantly, miR‐181a‐5p level markedly decreased in NK cells from PBMC of elderly people. Thus, our results demonstrated that the miRNAs profiles in NK cells change with aging, the decreased level of miR‐181a‐5p contributes to the defective NK cell development and function with aging. This opens new strategies to preserve or restore NK cell function in the elderly.

## Graphical abstract

MicroRNAs (miRNAs) regulate gene expression and thereby influence cell development and function. Deep sequencing of R1 and R2 NK cells revealed that the miRNAs profiles in NK cells change with aging. MiR‐l8la‐5p expression decreases in aged NK cells, and the decreased level of miR‐181a‐5p contributes to the defective NK cell maturation and function with aging.

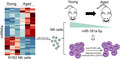

## 1 INTRODUCTION

MicroRNAs are a class of short (~22nt), endogenously initiated non‐coding RNAs, which post‐transcriptionally control gene expression via either translational repression or mRNA degradation (Cai et al., 2009). Each miRNA may suppress multiple mRNA targets, while one mRNA can be targeted by several miRNAs for precise control of multiple cellular processes, such as cell differentiation, proliferation, apoptosis, tumorigenesis, and host–pathogen interactions (Huang et al., 2011). MicroRNAs are crucial post‐transcriptional regulators of the immune system (Contreras & Rao, 2012), by negatively regulating the expression levels of important genes to significantly influence the development and function of the immune cells (Mehta & Baltimore, 2016).

Natural killer (NK) cells are crucial innate effector cells serving as the first line of defense against certain infectious pathogens and tumors due to their ability to rapidly release inflammatory cytokines and kill infected or transformed cells (Vivier et al., 2011). Multiple studies have shown that NK cells might be a promising cancer immunotherapeutic for many malignancies (Abel et al., 2018; Kim et al., 2019). NK cells develop in BM from the hematopoietic stem cells (HSCs). Firstly, HSCs develop into the common lymphoid progenitor (CLP), which is characterized by expression of IL‐7Rα (CD127), c‐kit (CD117), Sca‐1, and Flt‐3(CD135), as well as the lack of lineage markers such as CD3, CD4, CD8, CD19, Ter119, Gr‐1, and NK1.1. The cellular origin of NK cells in humans and mice can be traced back to oligopotent CLP. Secondly, the early progenitors develop into pre‐NK cell precursors (pre‐NKPs), which are defined as CD244^+^ c‐kit^low^ IL‐7Rα^+^ Flt‐3^−^ CD122^−^ and lineage negative. From the pre‐NKPs, cells develop into NK cell precursors (NKP), which are defined by expression of the IL‐15 receptor β chain (CD122), and lack of common lineage markers, including the NK cell markers NK1.1 and DX5 (CD49b). Then, the NKPs develop into the immature NK (iNK) population. Finally, iNK developed into the mature NK (mNK) cells. NK cells that have reached terminal maturation are fully functional (Geiger & Sun, 2016). In mouse, NK cells can be divided into four subsets according to surface expression of CD27 and CD11b: R0 NK cells (CD27^−^ CD11b^−^), R1 NK cells (CD27^+^ CD11b^−^), R2 NK cells (CD27^+^CD11b^+^), and R3 NK cells (CD27^−^CD11b^+^), that represent NK cells at distinct developmental stages. R0 and R1 NK cells are immature, R2 and R3 NK cells are mature NK cells, with R3 NK cells are terminally differentiated (Chiossone et al., 2009; Fang et al., 2008; Hayakawa & Smyth, 2006).

Multiple factors regulate the development and maturation of NK cells. Most studies of the molecular events that regulate NK cell development and function have focused on transcription factors and key signaling pathways (Kee et al., 2020; Wang & Malarkannan, 2020). Currently, miRNA studies in NK cells mainly focus on identifying those miRNAs that either regulate NK cell development or effector functions (Leong et al., 2014). Several miRNAs have been found to promote miR‐181 (Cichocki et al., 2011), miR‐150 (Bezman et al., 2011), or repress miR‐483 (Ni et al., 2013), miR‐583 (Yun et al., 2014) NK cell development. NK cells mediate functional responses through releasing apoptosis‐inducing granule proteins and producing effector cytokines such as IFN‐γ and TNF‐α. To date, several miRNAs, including miR‐155 (Zawislak et al., 2013), miR‐150 (Kim et al., 2014), miR‐181 (Cichocki et al., 2011), and miR‐29 (Ma et al., 2011), miR‐27a‐5p (Kim et al., 2011), miR‐30c‐3p (Gong et al., 2012), miR‐30e (Wang et al., 2012), and miR‐378 (Wang et al., 2012) have been indicated to play a role in regulating NK cell effector functions.

Our previous studies demonstrated that the proportion of R3 NK cells are significantly decreased in aged mice, indicating defective NK cell development with aging (Fang et al., 2010). The impaired NK cell development results in reduced effector functions of NK cells and susceptibility to poxvirus or influenza A virus infection in the aged mice (Beli et al., 2011; Fang et al., 2010). Previous studies have demonstrated that aging differentially affects NK cell subsets and NK cell function (Manser & Uhrberg, 2016). However, whether miRNAs are involved in this process is currently unknown.

In this study, we investigated the miRNA profiles of R1 and R2 NK cells from young and aged mice and identified differentially expressed miRNAs between young and aged NK cells.

## 2 RESULTS

### 2.1 Differential miRNA expression profiles of R1 and R2 NK cells between young and aged mice

Our previous studies showed impaired NK cell development in mice with aging (Fang et al., 2010). We reproduced this results in the current study. As shown in Figure S1, compared with the young mice, the percent of R2 and R3 NK cells were dramatically decreased in the spleen, LN and BM of aged mice. On the contrary, the percent of R1 NK cells was increased in the spleen, LN, and BM of aged mice, while there was no significant difference in the percent of R0 NK cells between the young and aged mice. Thus, compared with the young mice, the maturation of NK cells in the aged mice was severely impaired.

**FIGURE 1:**
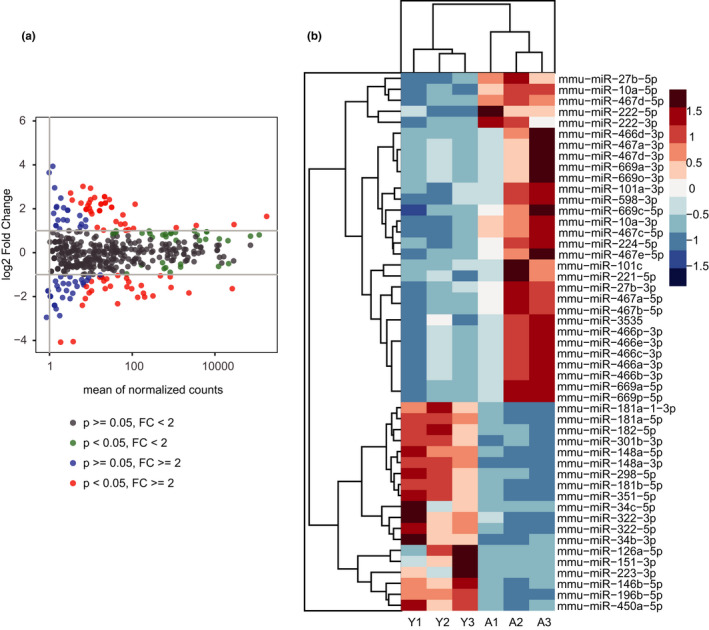
Differentially expressed miRNAs in CD27^+^ NK cells from young and aged mice. (a) The MA plot of differentially expressed miRNAs in CD27^+^ NK cells from young mice and aged mice. Each dot represents a differentially expressed miRNA in aged mice. The log2 fold change of each miRNA was calculated by the mean of normalized counts. P: *p*‐value; FC: fold change. (b) The heatmap of differentially expressed miRNAs profiled in CD27^+^ NK cells from the young mice (Y1–Y3 represent three individual repeats), and the aged mice (A1–A3 represent three individual repeats). The color scale shown on the left illustrates the relative expression levels of the miRNAs. The upregulated miRNAs are depicted in red color whereas the downregulated miRNAs are depicted in blue color

To study the mechanisms of the impaired NK cell maturation in the aged mice, we sorted the R1 and R2 NK cells (CD27^+^ NK cells) from the BM of young and aged mice. Deep sequencing revealed the miRNA profiling of CD27^+^ NK cells in the young and aged mice. Differential expression analysis was performed to identify differentially expressed miRNAs between young and aged mice. MicroRNAs that displayed a twofold or greater difference in expression level between young and aged mice were considered as differentially expressed, and lower values were not considered. A total of 49 miRNAs (from known miRNAs) displayed a twofold or greater difference in expression level. Among these, 30 miRNAs were upregulated and 19 miRNAs were downregulated in the aged NK cells compared with the young NK cells (Figure 1a,b). Those 49 miRNAs were further analyzed in heat map, 10 miRNAs were found to be highly consistent in three individual repeats and significantly different between young and aged NK cells (Figure 1b). Thus, we chose the 10 miRNAs as the candidate miRNAs.

### 2.2 Potential targets and functions of candidate miRNAs

**FIGURE 2:**
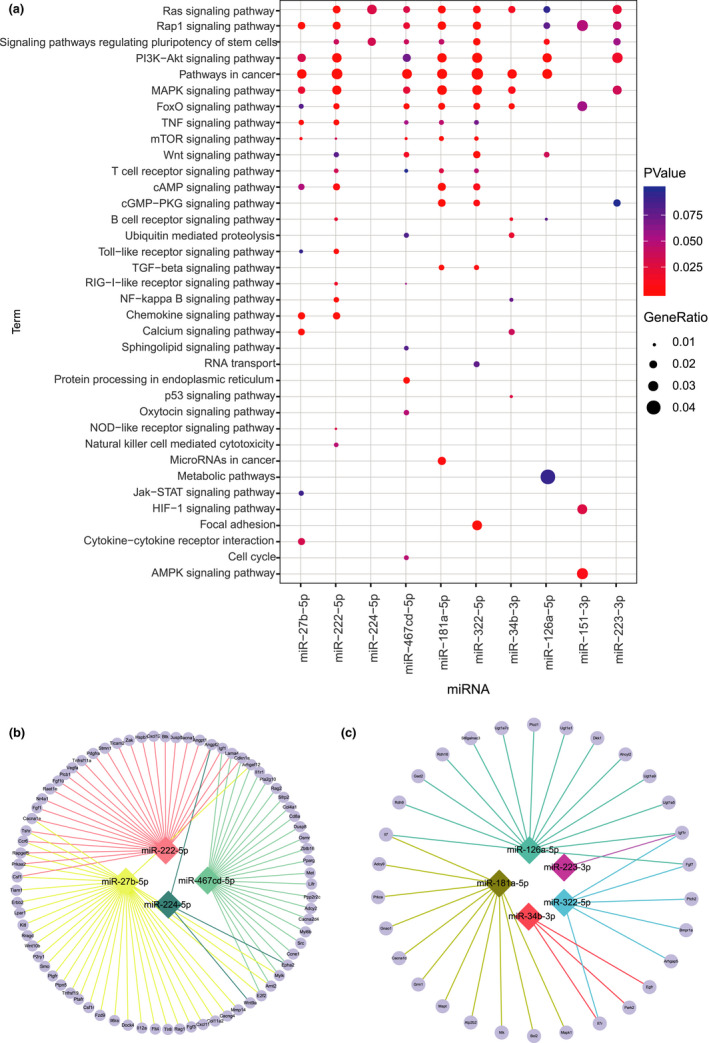
Potential targets and function analysis of the 10 candidate miRNAs. (a) The function of miRNA was analyzed based on the target gene information by KEGG pathway enrichment analysis. The abscissa represents miRNA, and the ordinate represents enriched function. Nodes represent enrichment extent. (b) Interactive network diagram of the four upregulated miRNAs in CD27^+^ NK cells from aged mice and their downregulated potential target genes. (c) Interactive network diagram of the five downregulated miRNAs in CD27^+^ NK from aged mice and their upregulated potential target genes. Rhombic dots represent the differentially expressed miRNAs. Round dots represent the differentially expressed target genes

Among the 10 candidate miRNAs, four miRNAs (miR‐27b‐5p, miR‐222‐5p, miR‐224‐5p, and miR‐467 cd‐5p) were upregulated in CD27^+^ NK cells of the aged mice; six miRNAs (miR‐181a‐5p, miR‐322‐5p, miR‐34b‐3p, miR‐126a‐5p, miR‐151‐3p, and miR‐223‐3p) were downregulated in CD27^+^ NK cells of the aged mice. To further identify the functions of these miRNAs, we predicted their potential target genes using Targetscan and analyzed the functions of these target genes. KEGG pathway analysis showed that the targets were significantly mapped in 36 pathways, including Ras, Rap1, PI3 K‐Akt, and MAPK signaling pathways (Figure 2a).

To further confirm the functions of potential targets regulated by the miRNAs, we performed mRNA profile analysis of the CD27^+^ NK cells from the young and aged mice. mRNAs that displayed a twofold or greater difference in expression level between young and aged NK cells were considered as differentially expressed. We conducted the miRNA‐target network analysis by combining the predicated target mRNAs and the differentially expressed mRNAs. For the upregulated miRNAs in the aged NK cells, we conducted the miRNA‐target network in Figure 2b. Among the predicted targets, transcription factor gene E2F2 was targeted by both miR‐27b‐5p and miR‐467 cd‐5p; chemokine gene CCR6 and colony‐stimulating factor gene CSF1(colony‐stimulating factor 1) were targeted by both miR‐27b‐5p and miR‐222‐5p. Among the downregulated miRNAs in the aged NK cells, we did not find differentially expressed mRNAs in the predicated targets of miR‐151‐3p. For the other five downregulated miRNAs, both miR‐181a‐5p and miR‐126a‐5p targeted IL‐7; miR‐181a‐5p targeted adenylate cyclase 9 (ADCY9), nemo like kinase (NLK), apoptosis regulator BCL2, and mitogen‐activated protein kinase 1(MAPK1). Other miRNAs targeted phosphatase genes and cancer genes (Figure 2c).

### 2.3 MiR‐181a‐5p and miR‐223‐3p both decline in the aged NK cells

**FIGURE 3:**
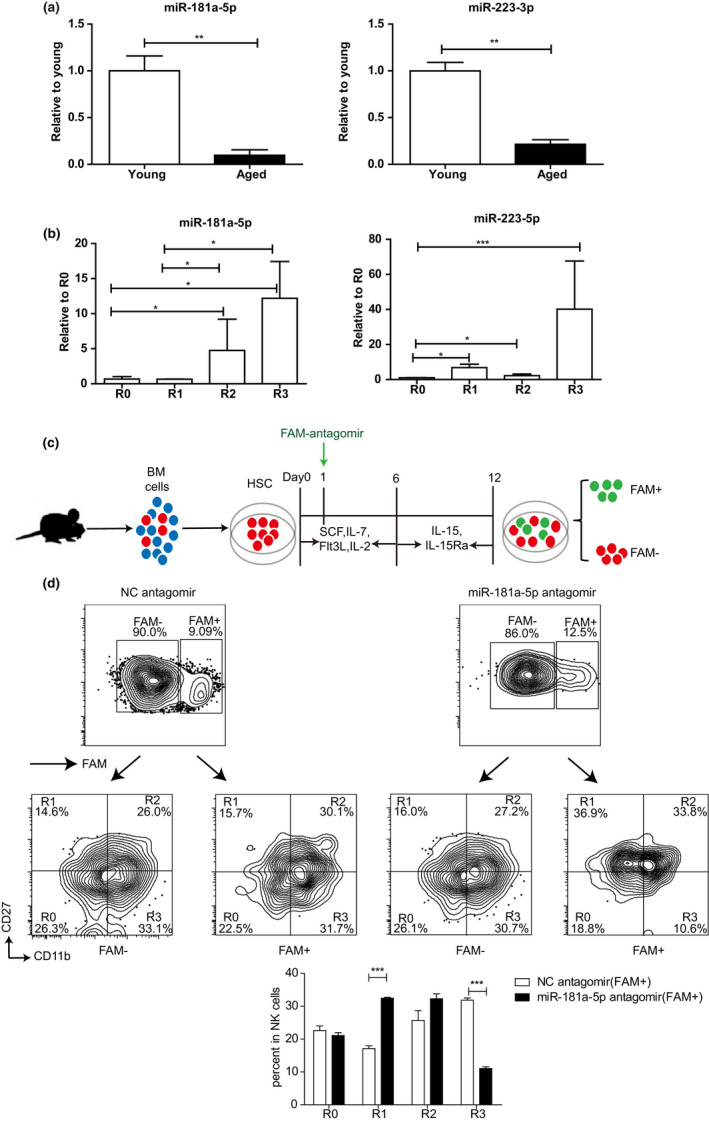
The declined expression of miR‐181a‐5p influences the maturation of NK cells in aged mice. (a) The expression levels of miR‐181a‐5p (left) and miR‐223‐3p (right) in CD 27^+^ NK cells of young or aged mice were detected using qRT‐PCR. The cells were sorted from bone marrow of the young and aged mice by using a FACSAria II (BD Biosciences) with purities >98%. (b) The expression levels of miR‐181a‐5p (left) and miR‐223‐3p (right) in different NK cell subsets of young mice. The cells were sorted from spleen, peripheral lymph nodes and bone marrow of the young mice. (c) Schematic of in vitro mouse NK cell differentiation using a two‐step cell culture system as described in Materials and Methods. HSCs purified from bone marrow of young CD45.1 mice were transfected with FAM‐labeled miR‐181a‐5p antagomir or FAM‐labeled NC antagomir. (d) Flow cytometry (above) and statistical results (below) of R0, R1, R2 and R3 NK cells in (c). Data were from three independent experiments (mean ± SEM). *p < 0.05, **p < 0.01, ***p < 0.001

Previous studies have shown that miR‐181 and miR‐223 are involved in regulating the development and function of NK cells (Leong et al., 2014). miR‐181a‐5p is one of the most well‐studied members of the miR‐181 family, which include four highly conserved mature miRNAs: miR‐181a, b, c and d (Cichocki et al., 2011; Li et al., 2015). In this study, we used qRT‐PCR to verify the expression of miR‐181a‐5p and miR‐223‐3p in CD27^+^ NK cells sorted from the BM of the young and aged mice. Significantly decreased expressions of miR‐181a‐5p and miR‐223‐3p were found in the aged NK cells (Figure 3a), which were consistent with the results of RNA sequencing. To further determine the miRNAs levels during the maturation of NK cells from R0 to R3, qRT‐PCR was performed in the sorted R0, R1, R2, and R3 NK cells from mouse spleens. As shown in Figure 3b, with the maturation of NK cells, the transcript of miR‐l8la‐5p increased, with the highest expression level in R3 NK cells. The highest expression level of miR‐223‐5p was also in the R3 NK cells. Our study demonstrated that the expression levels of mi‐181‐5p and miR‐223‐5p in NK cells both declined in the aged mice, and the highest expression level of miR‐l8la‐5p and miR‐223‐5p were both in the terminally matured R3 NK cells.

### 2.4 miR‐181a‐5p promotes the maturation of NK cells in vitro and in vivo

To study whether the above changed miRNAs affect the development and maturation of NK cells, we chose miR‐181a‐5p for further research. We established a system to imitate the in vitro development of NK cells on the basis of the study by Yin et al. (2015). We used FAM labeled‐miR‐181a‐5p antagomir to knock down miR‐181a‐5p, which can easily transfect cells. An antagomir from Caenorhabditis elegans (cel‐miR‐67‐3p) was used as a negative control (NC). The hemopoietic stem cells (HSC) isolated from BM of young mice were treated with miR‐181a‐5p antagomir or NC antagomir and cultured for 12 days as schematized in Figure 3c. Then, we tested the development and maturation of NK cells. We found that transfection of NC antagomir had no effect for the maturation of NK cells. However, the R1 NK cells were markedly increased, while R3 NK cells were significantly decreased in the miR‐181a‐5p antagomir treated group (FAM^+^) compared with the NC antagomir treated group (FAM^+^), respectively (Figure 3d). Thus, NK cell development in vitro was impaired with knockdown of miR‐181a‐5p.

**FIGURE 4:**
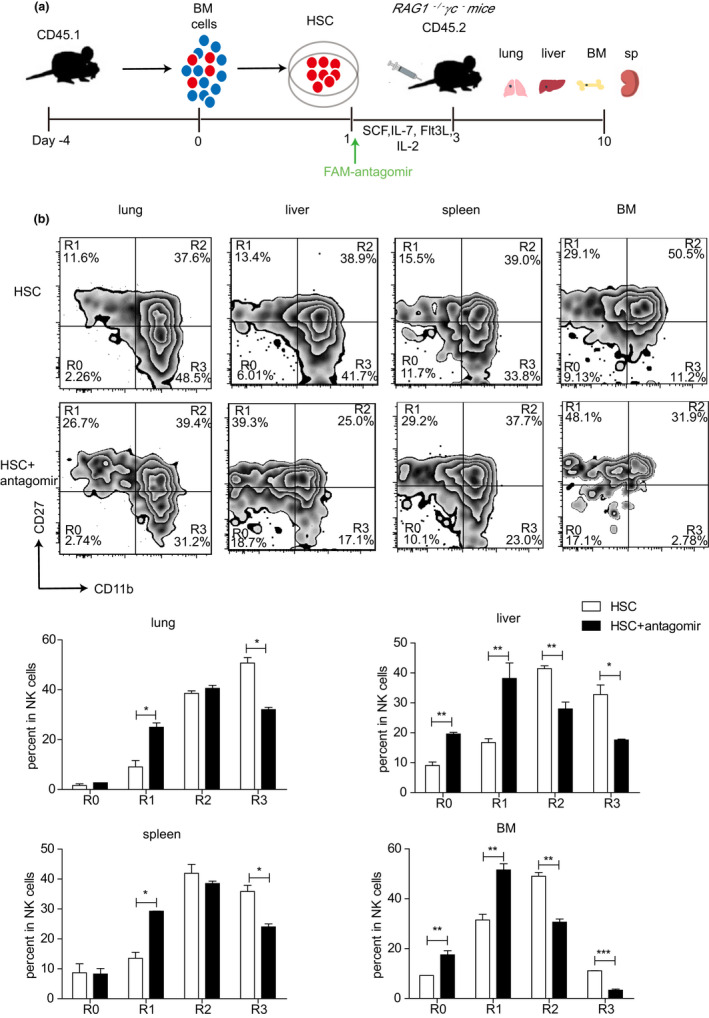
MiR‐181a‐5p is important for the maturation of NK cells in vivo. (a) Schematic of in vivo mouse NK cell differentiation using an adoptive transfer system as described in Materials and Methods. (b) Flow cytometry (above) and statistical results (below) of R0, R1, R2, and R3 NK cells in the lung, liver, spleen, and BM of the recipient mice. Data represented three independent experiments (mean ± SEM). *p < 0.05, **p < 0.01, ***p < 0.001

To further confirm the role of miR‐181a‐5p in the maturation of NK cells, we set up the in vivo system using the *RAG1* ^−/−^ *γc* ^−^ mice. HSCs isolated from BM of young mice were treated with antagomir for 48 h, then transferred into the *RAG1* ^−/−^ *γc* ^−^ mice to allow further development in vivo for another 7 days (Figure 4a). We analyzed the percentages of R0, R1, R2, and R3 NK cells in lung, liver, spleen, and BM of the recipient mice. Compared with the control, miR‐181a‐5p antagomir treated group exhibited a significant reduction of R3 NK cells in the lung, spleen, liver, and bone marrow. Meanwhile, the proportion of R1 NK cells in lung, liver, spleen, and BM were increased accordingly (Figure 4b). These results were resembled with the NK cell development in the aged mice (Figure S1), in which the miR‐181a‐5p level was declined. These results further demonstrated that the reduced miR‐181a‐5p inhibited the NK cell development and maturation in vivo, and the critical requirement for miR‐181a‐5p in NK cell development was cell intrinsic.

### 2.5 NLK and BCL2 are targets of miR‐181a‐5p in NK cells

**FIGURE 5:**
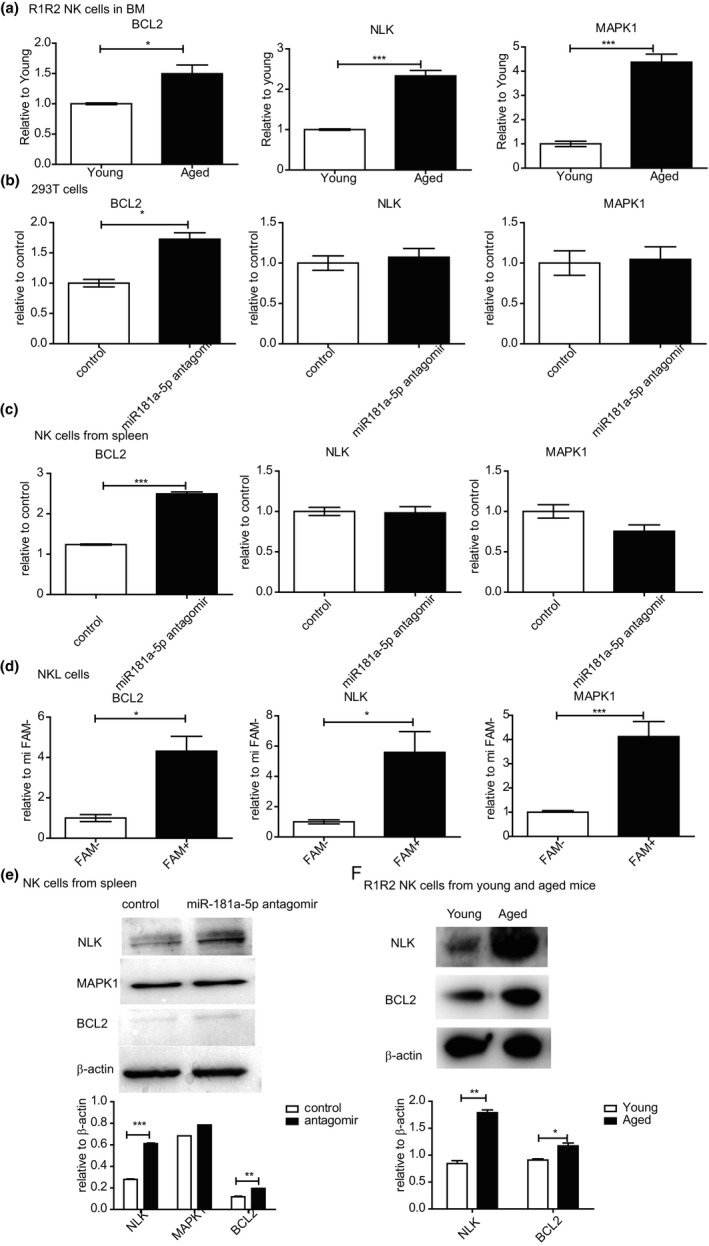
BCL2 and NLK are the target genes of miR‐181a‐5p. (a) CD27^+^ NK cells were sorted from bone marrow of young and aged mice. The transcripts of the potential target genes (BCL2, NLK, and MAPK1) were detected by qRT‐PCR. (b) 293 T cells and (c) NK cells purified from spleen of the young mice were transfected with miR‐181a‐5p antagomir with transfection efficiency >60%. The transcripts of the potential target genes were detected by qRT‐PCR. (d) NKL cells were transfected with miR‐181a‐5p antagomir. The FAM^+^ cells and FAM^−^ cells were separately sorted. The transcripts of the indicated genes in the FAM^+^ or FAM^−^ groups were detected by qRT‐PCR. Western blotting and grayscale analysis of the expression of potential target genes in the NK cells from mice spleen (e) or sorted BM R1 and R2 NK cells from young or aged mice (f). Data represent three independent experiments (mean ± SEM). *p < 0.05, ***p < 0.001

The miRNAs control gene expression via either translational repression or mRNA degradation. To study the molecular mechanisms, we further investigated the target genes of miR‐181a‐5p in regulating the maturation of NK cells in aged mice. We used Targetscan database and miRDB database to predict potential targets for miR‐181a‐5p. 674 potential targets were found for miR‐181a‐5p. We searched this list for genes with potential relevance to NK cell development and identified three target genes (BCL2, NLK, and MAPK1) that had been reported to be involved in NK cells development and function (Table 1). We verified the mRNA levels of these three genes in R1 and R2 NK cells of BM from both young and aged mice. As shown in Figure 5a, the transcripts of all the three target genes were significantly increased in aged mice (Figure 5a), which were concordant with the reduced expression of miR‐181a‐5p in aged mice (Figure 3a).

**TABLE 1:** The potential targets of miR181 that related to the NK cell development and function

miRNA	Target gene	References
miR‐181	NLK	Cichocki et al. (2011)
miR‐181	BCL2	Ouyang et al. (2012)
miR‐181a	MAPK1	Huang et al. (2016)

To further confirm that BCL2, NLK, and MAPK1 are the target genes of miR‐181a‐5p, we knocked down miR‐181a‐5p in 293 T cells using antagomir and measured the expression of BCL2, NLK, and MAPK1 by qRT‐PCR, respectively. Knockdown of miR‐181a‐5p in 293 T cells only resulted in significant increase of BCL2 transcript (Figure 5b). Then, we knocked down miR‐181a‐5p in purified NK cells from spleens of young mice using miR‐181a‐5p antagomir and obtained similar results as with that of 293 T cells (Figure 5c). miR‐181a‐5p is highly conserved in human and mouse. To further investigate the target genes of miR‐181a‐5p, we knocked down miR‐181a‐5p in human NK cell line NKL cells using antagomir and measured the transcripts of BCL2, NLK, and MAPK1. In NKL cells, knockdown of miR‐181a‐5p led to significantly increase in mRNA levels of NLK, BCL2, and MAPK1 (Figure 5d).

To verify the above results, protein levels of the NLK, BCL2, and MAPK1 were measured using Western blot. Knockdown of miR‐181a‐5p resulted in a significant increase in NLK and BCL2 protein levels in sorted NK cells from spleen, while the expression of MAPK1 remained at a similar level (Figure 5e). We further tested the protein levels of NLK and BCL2 in sorted R1 and R2 NK cells from young and aged mice. As shown in Figure 5f, the expression level of both NLK and BCL2 were significantly higher in the R1 and R2 NK cells from aged mice than that of young mice. Therefore, both the mRNA and protein levels of NLK and BCL2 were inversely related to the miR‐181a‐5p level in R1 and R2 NK cells. Our data supported a role for miR‐181a‐5p in the negative regulation of NLK and BCL2 expression in NK cells.

### 2.6 miR‐181a‐5p regulates the function of NK cells

**FIGURE 6:**
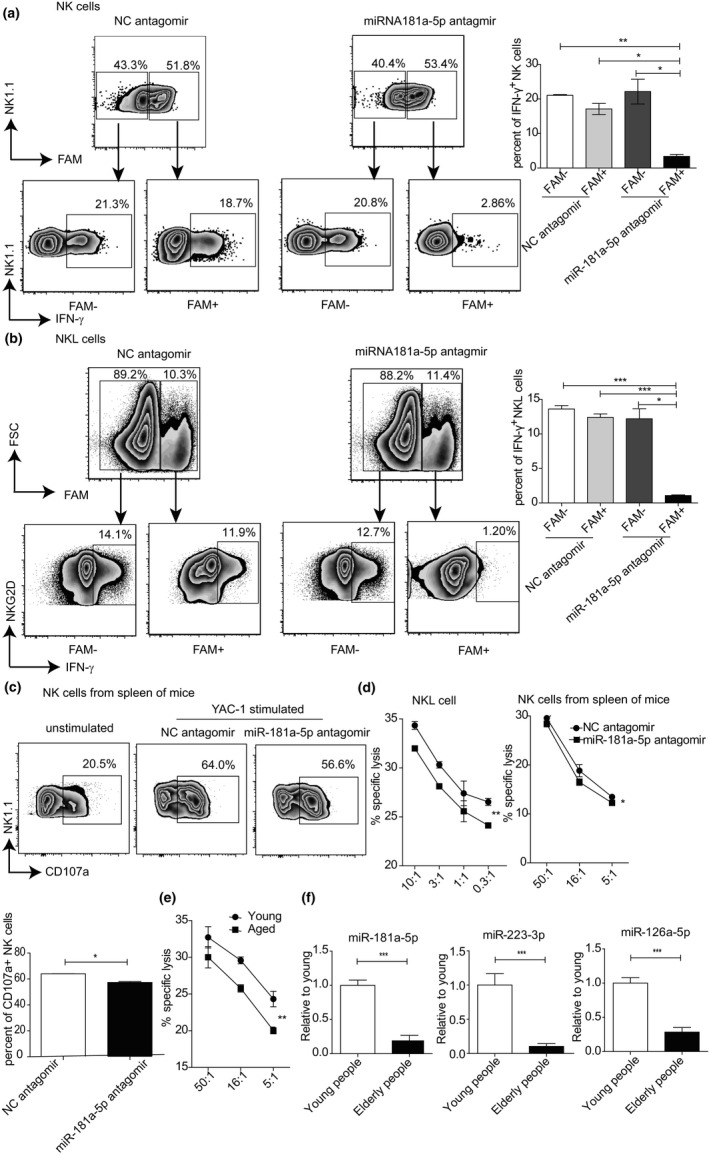
MiR‐181a‐5p regulates the function of NK cells. Splenocytes of the young mice (a) or NKL cells (b) were transfected with NC or miR‐181a‐5p antagomir, and stimulated by YAC‐1 or K562 cells, respectively. Representative flow cytometry plots (left) and statistical results (right) showing the IFN‐γ^+^ cells in gated NK1.1^+^CD3^−^ NK cells or NKL cells. (c) Splenocytes of the young mice were transfected with NC or miR‐181a‐5p antagomir, and stimulated by YAC‐1 cells. Representative flow cytometry plots (above) and statistical results (below) showing the frequency of CD107a^+^ cells in gated NK1.1^+^CD3^−^ NK cells. (d) NKL cells (left) or splenocytes of the young mice (right) were transfected with NC or miR‐181a‐5p antagomir. Lysis of target cells was shown in the summary graph (K562 cells (left); YAC‐1 cells (right)). (e) Lysis of YAC‐1 cells was shown. Splenocytes from young or aged mice were used as effectors. (f) NK cells were sorted from the PBMC of young and elderly people. The transcript level of miR‐181a‐5p, miR‐223‐3p, or miR‐126a‐5p was detected by qTR‐PCR. Data represent three independent experiments (mean ± SEM). *p < 0.05, **p < 0.01, ***p < 0.001

Previous studies have shown that miR‐181 regulates the secretion of IFN‐γ in NK cells (Leong et al., 2014). To further study the effect of miR181a‐5p on NK cell function, we treated NK cells from mouse spleen with miR‐181a‐5p antagomir or the NC antagomir, YAC‐1 cells were then added to the cell culture to stimulate NK cells. As shown in Figure 6a, the transfection efficiency of the antagomir in NK cells was nearly 50%. The percentage of IFN‐γ^+^ NK cells was similar between the FAM^+^ cells and the FAM^−^ cells in NC antagomir group. However, the percentage of IFN‐γ^+^ NK cells in FAM^+^ cells (3.39 ± 0.54%) of miR‐181a‐5p antagomir group was significantly decreased compared to that of the FAM^+^ cells in NC antagomir group (17.1 ± 1.6%) or the FAM^−^ cells (22.13 ± 3.58%) in miR‐181a‐5p antagomir group. We further treated NKL cells with miR‐181a‐5p antagomir or the NC antagomir. As shown in Figure 6b, the transfection efficiency of the antagomir in NKL cells was around 10%. The percentage of IFN‐γ^+^ cells was also similar between the FAM^+^ cells and the FAM^−^ cells in NC antagomir group. Again, the percentage of IFN‐γ^+^ NK cells in FAM^+^ cells (1.08 ± 0.09%) of miR‐181a‐5p antagomir group was significantly decreased compared with the FAM^+^ cells in NC antagomir group (12.4 ± 0.50%) or the FAM^−^ cells (12.2 ± 1.44%) in miR‐181a‐5p antagomir group. Thus, knockdown of miR‐181a‐5p reduced the production of IFN‐γ in stimulated NK cells from both mice and human.

NK cells are cytotoxic lymphocytes of the innate immune system that are capable of killing infected or cancerous cells. NK cells mediate their cytotoxic activity via two distinct pathways: release of cytotoxic granules or inducing death receptor‐mediated apoptosis (Prager & Watzl, 2019). Therefore, we further determined whether miR‐181a‐5p affects the cytotoxic activity of NK cells. Splenocytes from young mice were transfected with NC antagomir or miR‐181a‐5p antagomir, stimulated by YAC‐1 cells, and then stained for NK cell surface CD107a, a sign of degranulation. As shown in Figure 6c, the percentage of CD107a^+^ cells in miR‐181a‐5p antagomir group (55 ± 1.6%) was significantly lower than the NC antagomir group (62 ± 2.0%). Next, we analyzed the cytotoxicity of NK cells. K562 cells were used as the targets for NKL cells, and YAC‐1 cells were used as the targets for NK cells from spleen of mice, respectively. As shown in Figure 6d, both NKL cells and mice NK cells demonstrated reduced cytotoxicity to their target cells with miR‐181a‐5p antagomir treatment compared with the NC antagomir treatment. However, although there were statistical differences in both NK cell surface CD107a expression and cytotoxicity between the miR‐181a‐5p antagomir treated group and the NC group, the decreases were all small compared with the impact of miR‐181a‐5p antagomir on the NK cell production of IFN‐γ. Furthermore, the cytotoxic function of NK cells significantly decreased in the aged mice (Figure 6e), in which the miR‐181a‐5p level was declined (Figure 3a). In summary, our data indicated that miR‐181a‐5p was also important for the function of NK cells in mice and human.

### 2.7 miR‐181a‐5p level is decreased in the PBMC NK cells from elderly people

So far, our data have demonstrated that miR‐181a‐5p is important for the development and function of NK cells, and the expression level of miR‐181a‐5p decreases in mice NK cells with aging. To further test whether the expression level of miR‐181a‐5p also declines in human NK cells with aging, we isolated NK cells from PBMC of young and elderly people. Consistent with the observation in mouse, we observed a 75% reduction in the transcript levels of miR‐181a‐5p in NK cells from the elderly people compared with the young (Figure 6f). Thus, our results indicate that the declined miR‐181a‐5p expression is associated with impaired NK cell function with aging, both in mice and human.

Among the 10 candidate miRNAs in Figure 1b, four miRNAs (miR‐223‐3p, miR‐126a‐5p, miR‐151‐3p, and miR‐27b‐5p) were also highly conserved between human and mouse. Among them, we chose miR‐223‐3p and miR‐126a‐5p to investigate the levels of those miRNAs in human NK cells with aging. As shown in Figure 6f, the expression of miR‐223‐3p and miR‐126a‐5p were all decreased in the NK cells from the elderly people compared with the young, which was consistent with the results from mice. Thus, multiple miRNAs levels change in NK cells with aging, both in mice and human. However, whether each individual miRNA plays any role in the defective development and function of aged NK cells awaits further investigations.

## 3 DISCUSSION

NK cells are important immune cells, playing an important role in defense against infections and tumors. Thus, altered NK cell functionality with physiological aging has critical impact on overall immunity (Camous et al., 2012). This is of particular relevance because the elderly is more susceptible to infection, cancer, and other diseases. The contribution of the immune system to healthy aging and longevity remains largely unknown. A study of 108 immunologically normal elderly subjects indicated that the low NK cell activity is associated with the development of infections and death in the elderly people with impaired performance status (Ogata et al., 2001). Another study about lymphocyte activity in people of different ages found that the cytotoxic activities of NK cells are well preserved in the centenarians, suggesting that the well‐preserved NK cell activities likely contribute to their successful aging in centenarians (Sansoni et al., 1993). Therefore, keeping high performances of NK cells might be helpful for achieving healthy longevity in the elderly.

A broad range of miRNAs are known to regulate the development and function of NK cells. However, little is known about the association between miRNAs and the altered NK cell functionality with physiological aging. In this study, based on analysis of miRNomes, we found that the profiles of miRNAs in NK cells changed profoundly in mice with aging. MiR‐181a‐5p was one of those miRNAs, and was reduced in the aged CD27^+^ NK cells. By in vitro and in vivo experiments, we demonstrated that knockdown of miR‐181a‐5p intrinsically impaired the terminal maturation of NK cells. Therefore, the reduced level of miR‐181a‐5p may play a role in the defective maturation of NK cells with aging.

NK cells develop from BM‐derived HSCs and undergo terminal differentiation in both the bone marrow and various tissues, such as thymus, lymph node, and liver, as a means of generating functionally distinct and tissue‐specific NK cells. As shown in Figure S1, impaired NK cell development was observed in the spleen, LN, and BM, but not in the lung of aged mice, indicating that the impact of aging on NK cell maturation varies with different organs. In our adoptive transfer experiments, miR‐181a‐5p antagomir treatment resulted in a significant reduction of R3 NK cells in multiple organs including the lung of mice. This was different with the aged mice, albeit the reduction of R3 NK cells in lung was not as remarkable as other organs. The lung is responsible for gas exchange and in contact with the outside world. The unique environment in the lung might promote terminal NK cells differentiation as most lung NK cells are R3 NK cells. However, the main factors that drive NK cell differentiation in the lung deserve further researches.

Combined with mRNA sequencing, we identified that BCL2, NLK, and MAPK1 might be the targets of miR‐181a‐5p. The transcripts of all the three target genes were significantly increased in aged CD27^+^ NK cells, which were concordant with the reduced expression of miR‐181a‐5p. Furthermore, the protein level of BCL2 and NLK significantly increased in both mice spleen NK cells and BM CD27^+^ NK cells from the aged mice. Thus, our data suggested that reduced expression of miR‐181 influenced the terminal maturation of NK cells in the aged mice, partly through upregulation of NLK and BCL2. NLK is an inhibitor of Notch signaling and Notch signaling is important in NK cell development (Bachanova et al., 2009; Cichocki et al., 2011). MiR‐181 targets multiple Bcl‐2 family members (Ouyang et al., 2012), and BCL2 is required for the survival of NK cells (Viant et al., 2017). How miR‐181a‐5p influences the maturation of NK cells through BCL2 with the aged NK cells still needs more in‐depth research. Meanwhile, miR‐181a‐5p has diverse functions via distinct target genes in different cell types (Liang & Xu, 2020; Wen et al., 2020; Zhao et al., 2020). In our research, the putative target genes of miR‐181a‐5p antagomir were also varied in different cell types (293 T cells, NKL cells, or NK cells from spleen of mice).

MiR‐181a‐5p antagomir treatment dramatically reduced the production of IFN‐γ in NK cells, indicating that the cellular level of miR‐181a‐5p has a significant impact in NK cell secretion of IFN‐γ, which is consistent with previous report (Leong et al., 2014). In addition, miR‐181a‐5p antagomir treatment also resulted in a slight but statistically significant decrease in NK cell cytotoxic function. Furthermore, the cytotoxic function of NK cells was also decreased in the aged mice, which resembled the function of miR‐181a‐5p antagomir ex vivo. Thus, our data suggested that miR‐181a‐5p also regulates NK cell function, mainly on the production of IFN‐γ and also on the cytotoxicity. However, whether miR‐181a‐5p regulation of NK cell cytotoxicity has physiological significance awaits further investigations.

In human, NK cells can be divided into two subsets based on the expression level of CD56 and CD16. CD56^dim^CD16^+^ NK cells have high cytotoxic function, and CD56^bri^CD16^−^ NK cells have immune regulation function by producing cytokines (Melsen et al., 2016). During the development of human NK cells, CD56^bri^CD16^−^ NK cells subsequently differentiate into CD56^dim^CD16^+^ NK cells (Cichocki et al., 2019). A previous study reported that from CD56^bri^CD16^−^ NK cells to CD56^dim^CD16^+^ NK cells, the expression of miR‐181a‐5p was increased (Pesce et al., 2018), which was in accordance with our data that the terminally matured R3 NK cells in mice had the highest expression level of miR‐181a‐5p. Many studies reported that the function of NK cells was impaired in aged people (Witkowski et al., 2020). Our data showed that the expression of miR‐181a‐5p in NK cells from human PBMC declined with aging, indicating miR‐181a‐5p may be associated with the function changes of NK cells in the elderly.

Besides miR‐181a‐5p, there were other miRNAs changed in the NK cells during aging, such as miR‐223‐3p and miR‐126a‐5p (Figures 1b, 3a and 6f). These changes indicated that the functions of miRNAs are complicated in NK cells during aging. The molecular mechanisms underlying the maturation defect of NK cells with aging await further studies.

The cellular level of miRNA is easily to be regulated in experimental settings. Excitingly, recent innovations have made it possible to target and modulate miRNA in specific human cells as an avenue to correct or improve cell function. The successful development and clinical use of a messenger RNA (mRNA)‐based vaccines for SARS‐CoV‐2 have demonstrated the advantages of RNA‐based vaccine or therapeutic approaches. As miRNAs are key post‐transcriptional regulators, it is possible to development a lipid nanoparticle‐encapsulated miRNA with targeted cell delivery, for example, specifically deliver miR‐181a‐5p to NK cells to improve NK cell maturation and function in the elderly.

To our knowledge, this is the first report demonstrating the connection of changes of miRNAs to the developmental and functional defect of NK cells with aging. Our studies suggest that miRNAs might be used as potential treatment targets for regulating the development and function of NK cells with aging, which opens a new avenue for the mechanisms in immunosenescence and treatment for aging‐related disease. Furthermore, our researches provide ideas for improving the development and function of NK cells through miRNA regulation to improve healthy longevity for the elderly.

## 4 EXPERIMENTAL PROCEDURES

### 4.1 Ethics statement

The animal protocol used in this study was approved by the Research Ethics Committee of the Institute of Microbiology, Chinese Academy of Sciences (permit number APIMCAS2017034). All animal experimental procedures were performed in accordance with the Regulations for the Administration of Affairs Concerning Experimental Animals approved by the State Council of People's Republic of China. This study for human peripheral blood was performed with the informed consent of the participants. The experimental design and protocols used for human peripheral blood in this study were approved by the Research Ethics Committee of the Institute of Microbiology, Chinese Academy of Sciences (permit number APIMCAS2019056).

### 4.2 Cells culture and transfection

As standard tissue culture medium, we used complete RPMI or DMEM which consisted of the indicated tissue culture medium supplemented with 10% FCS, 100 IU/ml penicillin, 100 μg/ml streptomycin, 10 mM HEPES buffer (all from Gibco), and 0.05 mM 2‐ME (Amresco). Human embryonic kidney HEK293 T cells (ATCC, CRL‐3216), YAC‐1 cells (ATCC, TIB‐160), and K562 cells (ATCC, CCL‐243) were propagated in standard tissue culture medium. Human NK cell line NKL was a gift from Dr. Kerry S Campbell (Fox Chase Cancer Center, Philadelphia, PA). NKL cells were propagated in 1640 supplemented with 15% (v/v) fetal bovine serum (FBS) (Gibco), antibiotics (100 IU/ml penicillin and 25 mg/ml streptomycin), and recombinant human IL‐2 (200 U/ml). Splenocytes from mice were cultured in standard tissue culture medium with recombinant mouse IL‐2 (200 U/ml). All cells were grown at 37°C and 5% CO_2_.

FAM‐labeled miR‐181a‐5p antagomir or negative control antagomir (NC antagomir: cel‐miR‐67‐3p) were synthetized by Ribobio with the following sequences: mmu‐miR‐181a‐5p antagomir (sense, 5′‐ACUCACCGACAGCGUUGAAUGUU‐3′), NC antagomir (sense, 5′‐ UCACAACCUCCUAGAAAGAGUAGA‐3′). The 293 T cells, NKL cells, and splenocytes from mice were transfected with 200 nM miR‐181a‐5p antagomir or NC antagomir for 48 h in six‐well plates according to the manufacturer's protocols.

### 4.3 Mice

Young female C57BL/6 (B6, CD45.2) mice (7–9 weeks) were purchased from Vital River, China. Aged female B6 mice (17–20 months) were purchased from Vital River at 9 months old, and aged at animal facility under specific pathogen‐free (SPF) conditions. *RAG1* ^−/−^ *γc* ^−^ mice were generated as described (Yang et al., 2015). The CD45.1 C57BL/6 mice and *RAG1* ^−/−^ *γc* ^−^ mice were housed in an animal facility under SPF conditions.

### 4.4 Bone marrow NK cell purification and deep sequencing

For RNA sequencing, R1 and R2 NK cells (CD3^−^NK1.1^+^CD27^+^) in bone marrow (BM) of young B6 mice (six mice per group) or aged B6 mice (six mice per group) were sorted by using a FACSAria II (BD Biosciences) with purities >98%. Three individual experiments were performed to sort CD27^+^ NK cells. Then, the total RNA was isolated and deep sequencing was performed in the sequencing facility at Beijing Institute of Genomics.

### 4.5 Human PBMC isolation and NK cells purification

The human peripheral blood mononuclear cells (PBMC) from young (25–35 aged) and elderly people (>75 aged) were isolated with PBMC separation medium (TBD science, LTS1007) according to the manufacturer's instruction. The cells were washed twice with complete RPMI‐1640 medium. Then, the CD3^−^CD56^+^ NK cells were sorted using a FACSAria II (BD Biosciences) with purities >98%.

### 4.6 Quantitative real‐time PCR

Total RNA was extracted from sorted NK cells, 293 T cells, and NKL cells with TRIzol (Invitrogen). The miRNA was quantified using a TaqMan miRNA kit (Applied Biosystems) according to the manufacturer's protocols. The endogenous control U6 was used for normalization. The primers used were get from Ruibo. For mRNA, Universal Probe Library probes were purchased from Roche. Primers for mRNA were synthesized at Sangon Biotech. The primers used for mRNA were listed in Table 2. First‐strand cDNA was synthesized using oligo‐dT primers. qRT‐PCR was performed using a LightCycler 480 (Roche). The cycling conditions for RT‐PCR were as follows: 95°C for 10 min, followed by 40 cycles of 95°C for 10 s, 60°C for 30 s, and 72°C for 1 s. The fold increase in mRNA expression was determined using the ΔΔCt method relative to the values for the mock‐treated samples after normalization to glyceraldehyde 3‐phosphate dehydrogenase gene expression.

**TABLE 2:** Oligonucleotides and probes used for qPCR, related to the experimental procedures 4.6

Gene	Forward oligonucleotide	Reverse oligonucleotide	Probe
For mouse			
MAPK1	5′‐gacagagtacgtagccacacgtt‐3′	5′‐agcccacagaccaaatatcaat‐3′	50
BCL2	5′‐agtacctgaaccggcatctg‐3′	5′‐ggggccatatagttccacaaa‐3′	75
NLK	5′‐cacatactcaggggtcctcataa‐3′	5′‐agcaggtgaacagcttcatgt‐3′	20
GAPDH	5′‐tgtccgtcgtggatctgac‐3′	5′‐cctgcttcaccaccttcttg‐3′	80
For human			
MAPK1	5′‐agttcttgacccctggtcct‐3′	5′‐aacggctcaaaggagtcaaa‐3′	19
BCL2	5′‐agtacctgaaccggcacct‐3′	5′‐gccgtacagttccacaaagg‐3′	75
NLK	5′‐gatcaagccctataattagcttctca‐3′	5′‐acccacaacattggattttagac‐3′	9
GAPDH	5′‐agccacatcgctcagacac‐3′	5′‐gcccaatacgaccaaatcc‐3′	60

### 4.7 Hematopoietic stem cells isolation, and in vitro culture

Mouse hematopoietic stem cells (HSCs) were isolated from BM with the EasySep™ Mouse Hematopoietic Progenitor Cell Isolation Kit (stem cell), and cultured in complete 1640 medium supplemented IL‐7 (0.5 ng/ml; R&D systems), SCF (30 ng/ml; R&D systems), Flt3L (100 ng/ml; R&D systems), and IL‐2 (500 U/ml; R&D systems). After 24 h of culture, HSCs were transfected with 200 nM miR‐181a‐5p antagomir or NC antagomir in 96‐well plate. Three days later, fresh medium containing the above indicated cytokines was used to replace three quarters of the old medium, and continuing culture for another 3 days. Then, the culture medium was switched to complete 1640 medium supplemented with IL‐15 (30 ng/ml; R&D systems) and IL‐15 receptor α (120 ng/ml; R&D systems) and cells were further cultured for 6 days. Using this two‐step stromal cell‐free cell culture system, HSC cells developed into mature NK cells (Figure 4a).

### 4.8 Adoptive cell transfer

Donor mice (CD45.1) were treated with 5‐fluorouracil (5‐FU, 3 mg/mouse) for 4 day before bone marrow cells were harvested. HSCs expressing CD45.1 were isolated from BM with the EasySep™ Mouse Hematopoietic Progenitor Cell Isolation Kit (stem cell), and cultured in complete 1640 medium supplemented with IL‐7 (0.5 ng/ml; R&D systems), SCF (30 ng/ml; R&D systems), Flt3L (100 ng/ml; R&D systems), IL‐2 (500 U/ml; R&D systems), and 200 nM miR‐181a‐5p antagomir in 96‐well plate. After 48 h of culture, the HSCs were intravenously transferred into sublethally irradiated *RAG1* ^−/−^ *γc* ^−^ mice (expressing CD45.2). Seven days after transfer, recipient mice were euthanized and NK cells from the bone marrow, spleen, lung, and liver were analyzed by flow cytometry.

### 4.9 Isolation of lymphocytes

Lymphocytes from different organs were collected and processed individually. For spleen and BM, single‐cell suspensions were obtained by gentle mechanical dissociation in PBS containing 2% FBS. After hemolysis with 0.84% NH4Cl solution, cells were washed and resuspended in complete RPMI 1640 medium. The isolation of lung and liver lymphocytes was adapted as described previously (Lu et al., 2018). Briefly, the lung and liver were removed and passed through a cell strainer (BD Falcon) to obtain a single‐cell suspension. The cells were resuspended in 35% Percoll solution (in D‐Hank's buffer) and centrifuged at 830 *g* for 20 min at room temperature. The upper liquid phase was removed from the tube; the lymphocyte pellet was resuspended in 0.84% NH_4_Cl solution to lyse the red blood cells (RBCs) and then washed twice with PBS containing 2% FBS. Cells were washed and resuspended in complete RPMI medium.

### 4.10 Flow cytometric analysis

To determine NK cell maturation, up to 2 × 10^6^ lymphocytes were stained with surface antibodies at 4°C for 30 min. Detection of NK cell responses was performed as previously described (Lu et al., 2018). Briefly, 2 × 10^6^ splenocytes from mice (or NKL cells) were transfected with 200 nM miR‐181a‐5p antagomir or NC antagomir for 24 h and cultured at 37°C for 48 h in 96‐well plates. Then, 4 × 10^5^ YAC‐1 cells (or K562 cells when indicated) and anti‐CD107a antibody were added. After 3 h, brefeldin A was added to block the secretory pathway for the accumulation of IFN‐γ inside the cells. 2 h later, the cells were stained with surface antibodies, fixed, permeabilized, and stained for intracellular cytokines. The following anti‐mouse antibodies were used: anti‐CD3 (145‐2C11), anti‐NK1.1 (PK136), anti‐IFN‐γ (XMG1.2), anti‐CD27 (LG3A10), anti‐CD11b (M1/70), anti‐CD45.1 (A20), and anti‐ CD107a (clone: 1D4B). Cells were analyzed with a BD LSRFortessa flow cytometer (BD Biosciences).

### 4.11 Cytotoxicity analysis

NK cell cytotoxicity was analyzed using PKH26‐Annexin V‐7AAD staining method. Briefly, target cells (YAC‐1 cells for mice NK cells, or K562 cells for NKL cells) were labeled with PKH26 to distinguish from effector cells. Effector cells were splenocytes from young C57BL/6 mice or NKL cells. Target cell and effector cell were added to 96‐well plate at indicated E:T ratio (50:1, 16:1,5:1 for splenocytes and 10:1, 3:1, 1:1, 0.3:1 for NKL cells) and then incubated at 37°C for 4 h in 5% CO_2_ incubator. After incubation, cells were washed with pre‐cold PBS, stained with Annexin V‐APC and 7AAD Apoptosis Detection Kit (sungene biotech, ao2001‐11a‐ha‐h) and immediately analyzed by flow cytometer.

### 4.12 Western blot analysis

Detection of NLK, BCL2, or ERK2 (MAPK1) protein level was performed with rabbit anti‐NLK polyclonal antibody, rabbit anti‐BCL2 polyclonal antibody or rabbit anti‐ERK2 polyclonal antibody (all from Bioworld), respectively. Then, the bound Abs were detected using an HRP‐conjugated goat anti‐rabbit IgG secondary Ab (zhong shan jin qiao). Densitometric analysis was performed using an ECL Documentation system.

### 4.13 Statistical analysis

Statistical analysis was performed using Prism software (GraphPad). All statistical analyses were performed using an unpaired two‐tailed Student's *t* test or two‐way ANOVA test as applicable. When applicable, data were displayed as mean ± SEM.

## CONFLICT OF INTEREST

The authors declare no conflict of interests.

## AUTHOR CONTRIBUTIONS

J.L. and S.L. contributed to experimental design, experiments, data analysis, and manuscript preparation; X.L., W.Z., X.D., X.G., and J.X. contributed to experiments and data analysis; B.Y., L.S., Z.D., and L.X. contributed to experiment resources and materials, and data analysis; M.F. contributed to original project design, general supervision, experimental design, data analysis, and manuscript preparation.

## Supporting information

Figure S1

## Data Availability

This manuscript does not include large datasets.
