# Supplementary material for: Declined miR‐181a‐5p expression is associated with impaired natural killer cell development and function with aging
Source: Aging Cell. 2021 Mar 29;20(5):e13353. doi: 10.1111/acel.13353 (PMC8135006; doi:10.1111/acel.13353)
Supplement: Supplementary file 1 — Figure S1 [file ACEL-20-e13353-s001.pdf]

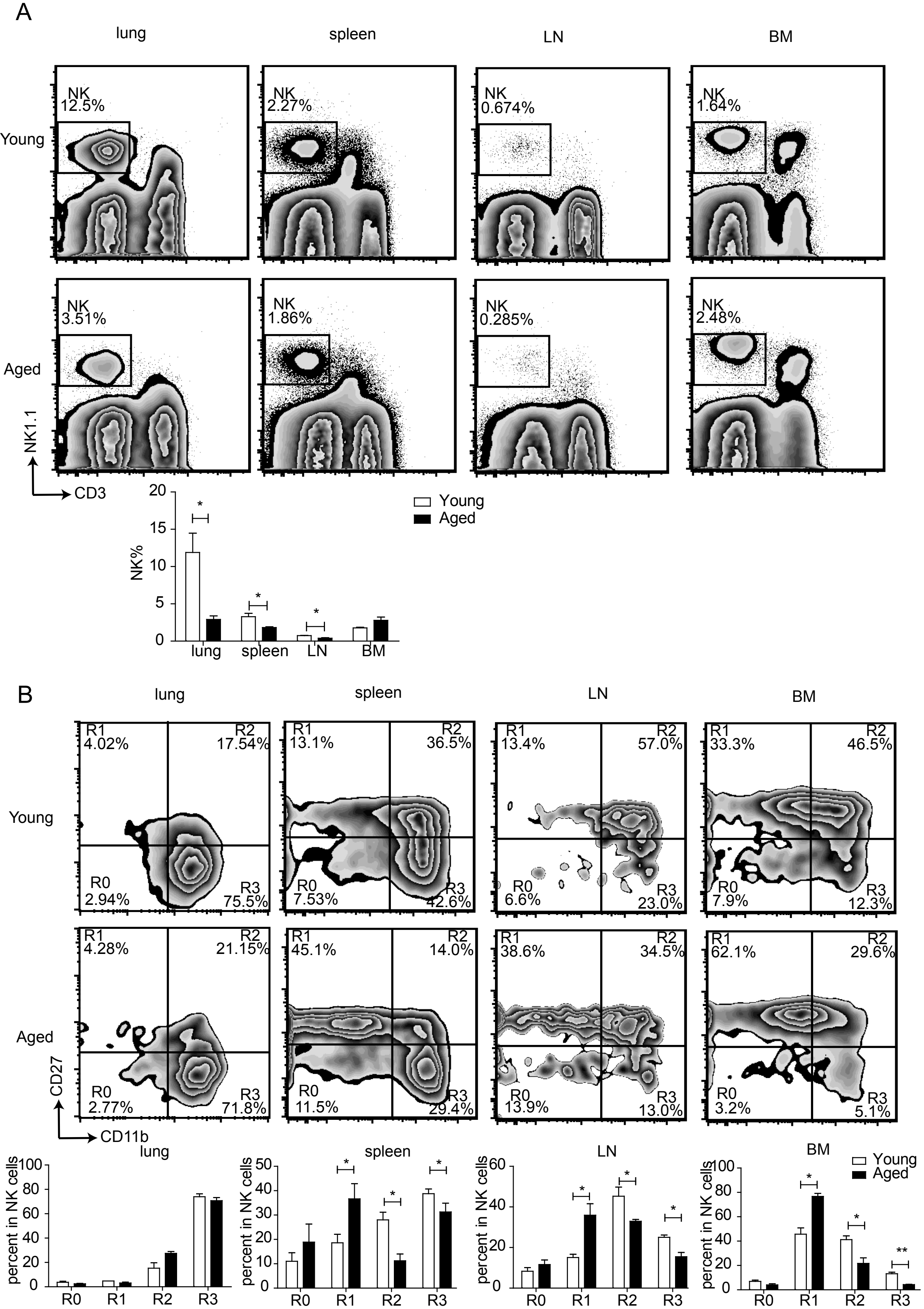

SUPPORTING FIGUREURE 1 The maturation of NK cells in the aged mice is severely impaired. (A) Flow cytometry and statistical results of the percent of NK cells in lung, spleen, LN and BM of the young and aged mice. (B) Flow cytometry and statistical results of the percent of R0, R1, R2, R3 NK cells in lung, spleen, LN and BM of the young and aged mice. *p < 0.05, **p <0.01.
